# Comparison of surgical and postoperative pregnancy outcomes between electrotomy and cold instruments for hysteroscopic myomectomy: a single-center, 3-year retrospective study

**DOI:** 10.1007/s00404-025-08131-2

**Published:** 2025-07-26

**Authors:** Panyu Chen, Danqi Shao, Weie Zhao, Yujie Li, Cong Fang, Lei Jia, Manchao Li

**Affiliations:** 1 Department of Reproductive Medicine Center, The Sixth Affiliated Hospital of Sun Yat-Sen University, Guangzhou, Guangdong China; 2 Department of Medical Ultrasonics, The Sixth Affiliated Hospital, Sun Yat-Sen University, Guangzhou, Guangdong China; 3 Guangdong Engineering Technology Research Center of Fertility Preservation, Guangzhou, Guangdong China; 4 Biomedical Innovation Center, The Sixth Affiliated Hospital, Sun Yat-Sen University, Guangzhou, Guangdong China

**Keywords:** Cold instruments, Electrotomy, Hysteroscopic myomectomy, Submucosal fibroids

## Abstract

**Purpose:**

To compare and analyze surgical outcomes and postoperative pregnancy outcomes between electrotomy and cold instruments for hysteroscopic myomectomy.

**Methods:**

This study included patients who were diagnosed with submucosal fibroids (FIGO 0-2) in our center and underwent hysteroscopic myomectomy from January 2022 to November 2024. Hysteroscopic myomectomy was performed by either bipolar system or cold instruments. Surgical and postoperative pregnancy outcomes were recorded and analyzed.

**Results:**

A total of 36 patients underwent hysteroscopic myomectomy at our center during the 3-year period, 21 in the cold instruments group and 15 in the electrotomy group. The mean age of the patients in the cold instruments group was significantly higher than that of the electrotomy group (39.00 ± 5.18 vs 35.20 ± 3.45, *P* = 0.019). In the electrotomy group, four patients found residual fibroids and required a second surgery. The incidence of residual fibroids and a second procedure were significantly higher than those in the cold instruments group (4/15(26.67%) vs 0, *P* = 0.023). The postoperative biochemical pregnancy rate and clinical pregnancy rate were higher in patients in the cold instruments group, but the difference was not statistically significant (14/21 (66.67%) vs 8/15 (53.33%), *P* = 0.644; 10/21(47.62%) vs 5/15(33.33%), *P* = 0.607).

**Conclusion:**

Cold instruments for hysteroscopic myomectomy seem to be a safe and feasible surgical procedure. It has an advantage over electrotomy in terms of complete removal of submucosal fibroids. Moreover, cold instruments for hysteroscopic myomectomy have no electrothermal damage to normal endometrium, which is favorable for pregnancy as soon as possible after surgery.

## What does this study add to the clinical work

**Table:** 

Cold instrument hysteroscopic myomectomy demonstrates superior complete fibroid removal compared to electrotomy, with no thermal endometrial damage, potentially enhancing postoperative pregnancy outcomes.	

## Introduction

Fibroids that grow inside or towards the uterine cavity are called submucosal fibroids, and the International Federation of Gynecology and Obstetrics (FIGO) classifies them as type 0, 1, and 2 based on their relationship to the uterine cavity [1]. Submucosal fibroids may impact the endometrial cavity, thereby plausibly impacting embryo implantation and development [2]. There is fair evidence that hysteroscopic myomectomy for submucosal fibroids improves clinical pregnancy rates. A prospective study by Maria Luisa Casini followed the pregnancy outcomes of 52 patients with submucosal fibroids, including 30 patients in the hysteroscopic surgery group, who tried to conceive on their own after 3 months of postoperative abstinence, and 22 patients who did not undergo surgery. Clinical pregnancy rate was significantly higher in patients with hysteroscopic removal of submucosal fibroids than in those who did not undergo surgery [3]. Similar conclusions were reached in the same study in patients with submucosal fibroids combined with intermural fibroids. Patients who underwent hysteroscopic myomectomy for submucosal fibroids had a significantly higher postoperative pregnancy rate than those who did not. A study by Pritts in 2009 showed that patients after hysteroscopic removal of submucosal fibroids achieved similar pregnancy outcomes to infertile patients with normal uterine cavities, and both groups had significantly higher clinical pregnancy rates than patients, especially women with myomas in situ (relative risk [RR] 2.03, CI 1.08–3.82, *P* = 0.028) [4].

Hysteroscopic removal of submucosal fibroids can be accomplished by electrotomy or non-thermal instruments. Surgical outcomes between unipolar and bipolar electrosurgery were compared in previous study, and there were no significant differences between them in terms of improvement in menstrual symptoms and postoperative pregnancy outcomes [5]. Bipolar electrosurgical systems are now more widely used because of their advantages in safety and operational efficiency. Previous study had been conducted to explore the results of the cold loop hysteroscopic myomectomy. A total of 1690 submucosal fibroids were resected in the study, with 1 to 5 fibroids being removed at each procedure. One thousand and seventeen of the one thousand two hundred fifteen patients only went through single procedure. There was a total of 12 intraoperative complications with an incidence of 0.84%. No cases of uterine perforation due to electrothermal injury or intravasation syndrome were reported [6]. Cold instruments hysteroscopy has been introduced in our center for the management of submucosal fibroids. Cold instruments include scissors, single-jointed Allis forceps, and double-jointed spoon forceps. No studies have been conducted on the management of submucosal fibroids with non-electrothermal energy devices such as cold scissors or forceps. The purpose of this study was to retrospectively compare the surgical results and postoperative pregnancy outcomes between electrotomy and cold instruments for hysteroscopic myomectomy in our center over the past 3 years.

## Methods

### Study design

We conducted a retrospective cohort study that included patients who were receiving IVF-ET (in vitro fertilization-embryo transfer) at our center and diagnosed as type 0–2 uterine fibroids according to FIGO standards from January 2022 to November 2024. All patients underwent a three-dimensional ultrasound before surgery. The three-dimensional ultrasound determines the type, size, and number of fibroids. Informed consent was obtained from all patients, and the research protocol was approved by the ethics committee of the Sixth Affiliated Hospital of Sun Yat-sen University (2017ZSLYEC-016S).

### Exclusion criteria

Patients with a combination of other uterine abnormalities were excluded, including uterine adhesion, uterine septum, uterine polyps, etc.

### Procedure

**Fig. 1:**
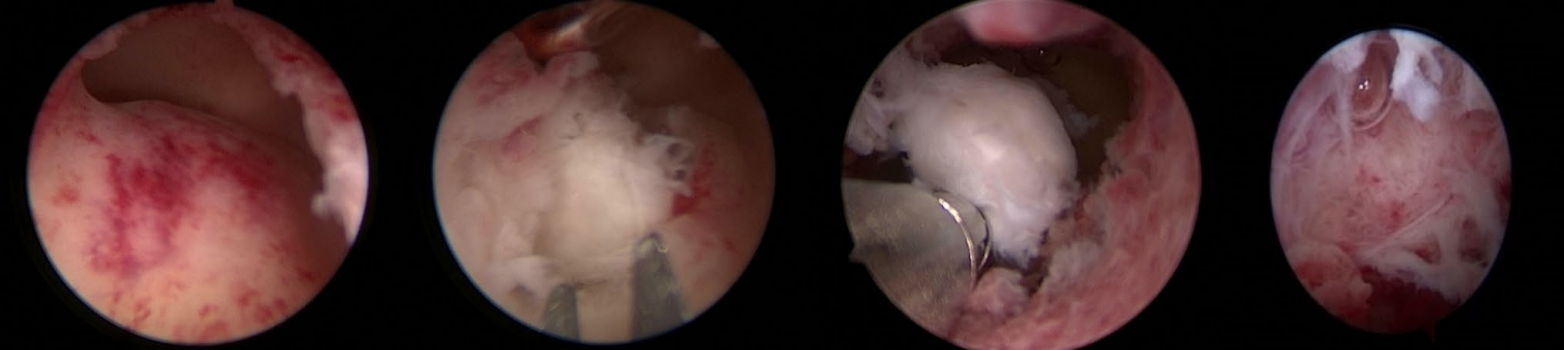
Intraoperative hysteroscopic picture of cold instruments for hysteroscopic myomectomy, using scissors and double-jointed spoon forceps

**Fig. 2:**
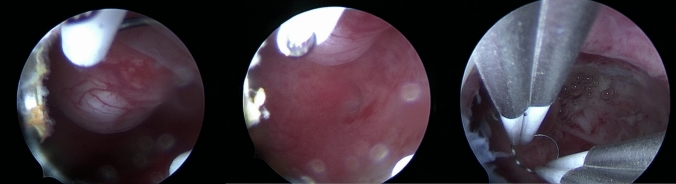
Intraoperative hysteroscopic picture of electrotomy for hysteroscopic myomectomy

All hysteroscopies and treatments were performed by experienced surgeons in our center with many years of clinical experience. All surgeons have the necessary qualifications and electrosurgical skills to perform hysteroscopic cold instruments and electrosurgical procedures. Hysteroscopy was performed 3–7 days after the end of the last menstrual period under general anesthesia. Saline was applied as distension media. Inflow pressure was set at 100 ~ 120 mmHg. The submucosal fibroids were separated using micro-scissors, single-jointed Allis forceps or double-jointed spoon forceps in the cold instruments group (Fig. 1) or excised using a bipolar dissection loop-shaped electrode in the electrotomy group (Fig. 2).

### Surgical parameters

Surgical parameters included the duration of the procedure, the amount of blood loss, the total amount of distension media, the amount of fluid deficit, the incidence of fluid overload, the incidence of residual fibroids, the need for a second procedure, and the presence of uterine adhesions postoperatively.

### Comparison of pregnancy outcomes

Postoperative pregnancy outcome parameters included the number of cycles experienced to achieve clinical pregnancy, type of assisted reproductive technology (ART) postoperatively, cycle type, cycle protocol, endometrium thickness and stage on the day of progesterone administration, embryo type, embryo grade, biochemical pregnancy rate, and clinical pregnancy rate.

### Statistical analysis

Statistical analysis was performed with R software (version 4.1.1) and IBM SPSS statistical software version 23 for Windows (IBM Corp., Armond, NY, USA). Quantitative variables that followed a normal distribution were expressed as mean and standard deviation (SD), and quantitative variables that did not follow a normal distribution were expressed as median and interquartile range (IQR). Qualitative variables were presented as counts and percentages, and the comparisons were performed by the Chi-square test or Fisher’s exact test, as appropriate. Values of *P* < 0.05 were considered to indicate statistical significance.

## Results

During the 3 years from 2022 to 2024, a total of 36 patients underwent hysteroscopic myomectomy at our center. Among them, 21 were in the cold instruments group and 15 were in the electrotomy group. The mean age of the patients in the cold instruments group was significantly higher than that of the electrotomy group, and the difference reached statistical significance (39.00 ± 5.18 vs 35.20 ± 3.45, *P* < 0.05). There was no statistically significant difference between the two groups in terms of ovarian reserve, type, volume, and number of submucosal fibroids (*P* > 0.05, Table 1).

**Table 1:** Comparison of baseline characteristics between electrotomy and cold instruments group

	Cold instruments	Electrotomy	*P*
*n*	21	15	
Age (mean (SD))	39.00 ± 5.18	35.20 ± 3.45	0.019
AMH (median (IQR))	1.58 [0.47; 2.27]	2.82 [0.77; 4.17]	0.205
*Type of fibroids*			0.076
FIGO I	5 (23.81%)	6 (40.00%)	
FIGO II	16 (76.19%)	7 (46.67%)	
Mixed	0	2 (13.33%)	
Volume of fibroids (cm^3^, median (IQR))	0.51 [0.12; 2.69]	0.51 [0.15; 4.80]	0.45
Number of fibroids (median (IQR))	1.00 [1.00; 1.00]	1.00 [1.00; 1.00]	0.364

*IQR* interquartile range

**Fig. 3:**
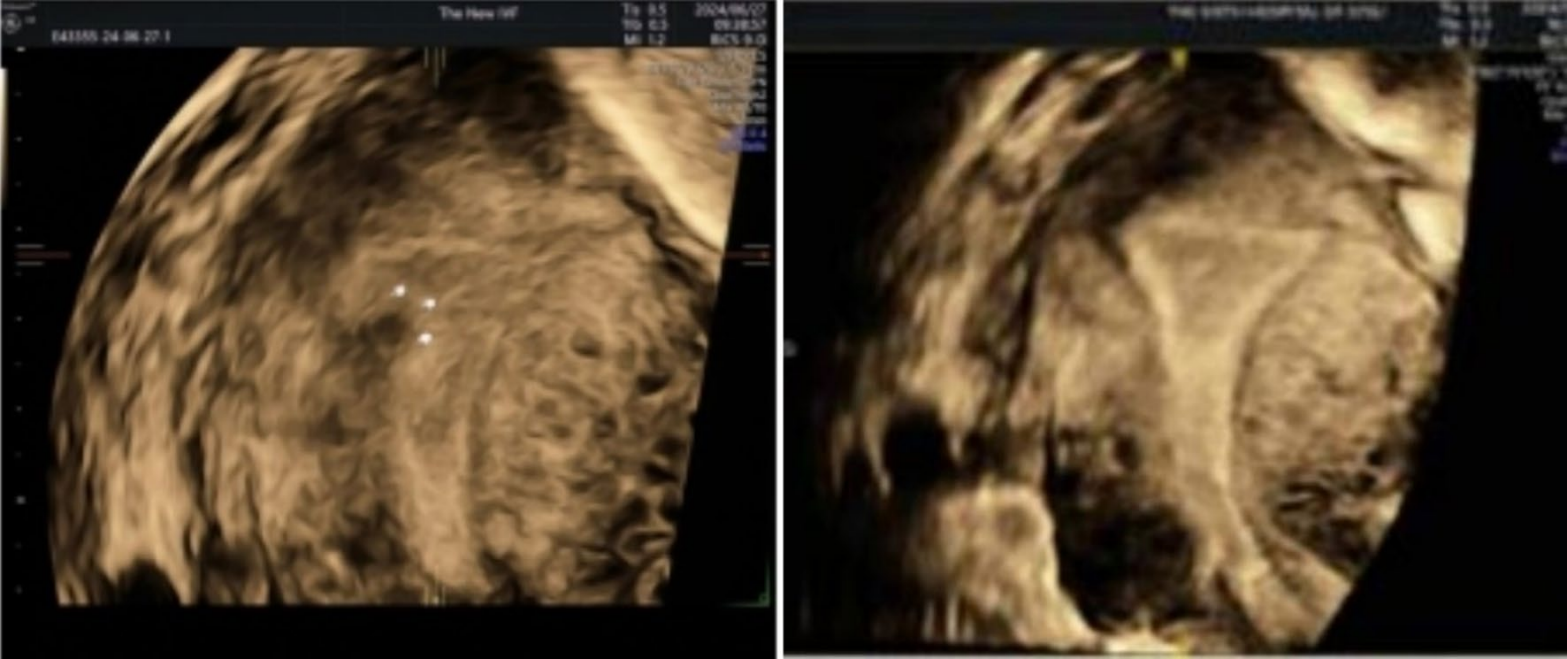
Three-dimensional ultrasound images of before and after cold instruments for hysteroscopic myomectomy

**Fig. 4:**
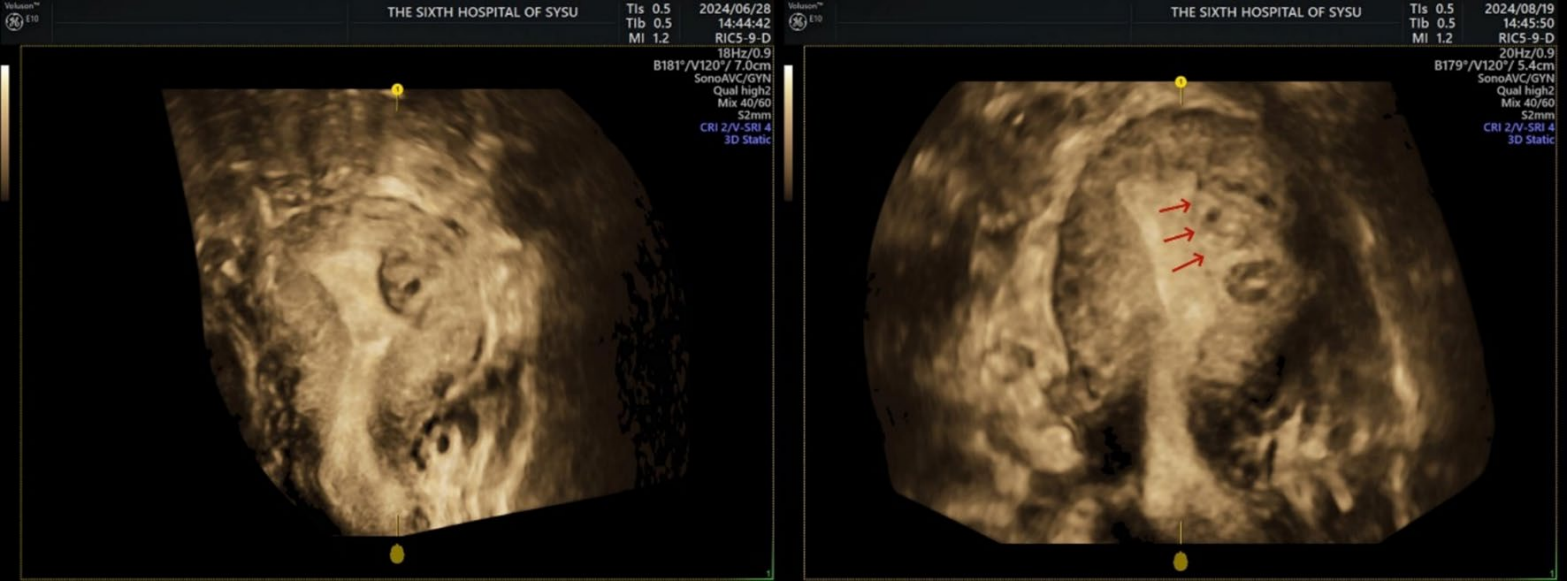
Three-dimensional ultrasound images of before and after electrotomy for hysteroscopic myomectomy, arrow pointing to residual fibroids

In the comparison of surgical parameters, patients in the cold instruments group underwent a longer duration of surgery, a greater total amount of distension media, a greater amount of fluid deficit. Patients in the electrotomy group had a greater amount of blood loss. However, none of the differences in the above indicators reached statistical significance. There were no fluid overload and uterine adhesions in both groups. A successful cold instrument for hysteroscopic myomectomy is shown in Fig. 3. Notably, 4 of the 15 patients in the electrotomy group found residual fibroids postoperatively and required a second surgery. The incidence was 26.67%, which was significantly higher than that of the cold instruments group (*P* < 0.05, Table 2; Fig. 4).

**Table 2:** Comparison of surgical parameters between electrotomy and cold instruments group

	Cold instruments	Electrotomy	*P*
*n*	21	15	
Duration of the procedure (min, median (IQR))	32.00 [23.00; 42.00]	23.00 [20.00; 31.00]	0.064
Amount of blood loss (ml, mean (SD))	4.33 ± 4.37	5.40 ± 6.09	0.544
Total amount of distension media (ml, median (IQR))	3000.00 [2200.00; 4700.00]	3000.00 [2900.00; 3650.00]	0.949
Amount of fluid deficit (ml, median (IQR))	500.00 [400.00; 700.00]	400.00 [200.00; 550.00]	0.168
Incidence of fluid overload	0	0	/
Incidence of residual fibroids	0	4 (26.67%)	0.023
Need for a second procedure	0	4 (26.67%)	0.023
Uterine adhesions postoperatively	0	0	/

*IQR* interquartile range

Postoperative pregnancy outcomes were compared between the two groups. Most patients in both groups achieved pregnancy during the first cycle of ART treatment postoperatively. The majority of patients underwent IVF and embryo transfer was performed in a frozen–thawed cycle. Patients in both groups had a favorable endometrial profile. The mean endometrial thickness on the day of progesterone administration was 9.55 ± 1.81 mm in the cold instruments group and 9.64 ± 2.13 mm in the electrotomy group. There was no difference in the type and grade of embryos transferred between the two groups. The biochemical and clinical pregnancy rates were higher in the cold instruments group, 66.67% and 47.62%, respectively, than in the electrotomy group, 53.33% and 33.33%. However, the difference did not reach statistical significance (*P* > 0.05, Table 3).

**Table 3:** Comparison of pregnancy outcomes between electrotomy and cold instruments group

	Cold instruments	Electrotomy	*P*
*n*	21	15	
*No. of cycles experienced to achieve clinical pregnancy*			1.000
1	9 (90.00%)	5 (100%)	
3	1 (10.00%)	0	
*Type of assisted reproductive technology postoperatively*			0.582
IUI	1 (4.76%)	0	
IVF	15 (71.43%)	9 (60.00%)	
ICSI	4 (19.05%)	6 (40.00%)	
PGT	1 (4.76%)	0	
*Cycle type*			1
Frozen–thawed	18 (85.71%)	14(93.33%)	
Fresh	1 (4.76%)	1 (6.67%)	
Mixed	1 (4.76%)	0	
*Cycle protocol*			0.469
HRT	5 (23.81%)	4 (26.67%)	
GnRHa + HRT	2 (9.52%)	2 (13.33%)	
GnRH antagonist	1 (4.76%)	0	
Follicular phase GnRHa	0	1 (6.67%)	
Mild stimulation	3 (14.29%)	2 (13.33%)	
Natural cycle	9 (42.86%)	6 (40.00%)	
Endometrium thickness on the day of progesterone administration (mm, mean (SD))	9.55 ± 1.81	9.64 ± 2.13	0.893
*Endometrium stage*			0.35
A	17 (85.00%)	12 (80.00%)	
A-B	1 (5.00%)	1 (6.67%)	
B	0	2 (13.33%)	
C	2 (10.00%)	0	
*Type of embryos transferred*			0.88
D3	8 (40.00%)	5 (33.33%)	
D5	10 (50.00%)	9 (60.00%)	
D6	2 (10.00%)	1 (6.67%)	
*Grade of embryos transferred*			1.000
Good quality	17 (85.00%)	13 (86.67%)	
Inferior quality	3 (15.00%)	2 (13.33%)	
Biochemical pregnancy rate	14 (66.67%)	8 (53.33%)	0.644
Clinical pregnancy rate	10 (47.62%)	5 (33.33%)	0.607

*IUI* intrauterine insemination, *IVF* in vitro fertilization, *ICSI* intracytoplasmic sperm injection, *PGT* preimplantation genetic testing, *HRT* hormone replacement therapy, *GnRHa* gonadotropin-releasing hormone agonist

## Discussion

The results of this study confirm the effectiveness of hysteroscopic cold instruments in complete removal of submucosal fibroids. A favorable postoperative pregnancy rate was also obtained due to its absence of electrothermal damage and protection of the normal endometrium.

Previous studies have compared the efficacy between thermal loop and cold loop in the treatment of submucosal fibroid. Mazzon conducted a retrospective analysis of 1434 submucosal fibroids surgeries. They used monopolar to remove submucosal fibroids, and after confirming that fibroids protruding into the uterine cavity had been removed, the remaining fibroids in the myometrium were scooped out using a specially designed cold loop. Fibroids were completely removed in a single operation in 1017 patients, and no uterine perforation or fluid overload was reported. Cold loop removal of submucosal fibroids better protected the normal endometrium [6]. A subsequent study by Sardo corroborated the efficacy of cold loop in the elimination of submucosal fibroids. Of the 72 patients with submucosal fibroids in this study, 70 of them had successful hysteroscopic myomectomy using cold loop without surgical complications. A notable finding of that study was that the small fovea that resulted from hysteroscopic myomectomy was almost restored by 6 weeks postoperatively [7]. Cold scissors are utilized to excise the surface endometrium of the fibroid, which provides a protective effect on the endometrium in patients with fertility requirements. Allis forceps or spoon forceps are then utilized to grasp and rotate the fibroids until it is completely detached. The procedure ensures complete removal of fibroids and seem to be highly effective in preventing residual fibroids. It also preserves the pseudo-capsule of the fibroid. Numerous studies have demonstrated that preserving the pseudocoelom during myomectomy results in a reduced incidence of early and late surgical complications and enhanced healing by preserving the integrity of the myometrium. This, in turn, promotes favorable pregnancy and delivery outcomes [8]. Furthermore, the uterine cavity demonstrates a rapid recovery following the cold instruments approach. Embryo transfers were successfully performed within the second month post-surgery. In addition, the thickness of the endometrium was maintained at approximately 9 mm, indicating that the cold instruments did not result in any damage to the endometrium or compromise fertility in the postoperative period [9].

Osorio et al. in Colombia, through a video, reported a case utilizing a newly designed double-lumen intracervical cannula that allowed the concomitant introduction of the Bettocchi diagnostic hysteroscope and a 5-mm laparoscopic tenaculum into the uterine cavity for complete non-fragmented fibroid extraction under direct visualization after blunt detachment of submucosal fibroids in 2021 [10]. The primary function of this instrument is to facilitate the surgical removal of fibroids under direct vision, thereby obviating the potential adverse effects of blind manipulation on the uterine cavity. In the cold instruments group of our study, spoon forceps or Allis forceps were utilized during hysteroscopy, once again achieving complete removal of fibroids under direct vision. It has been demonstrated that avoiding slicing the fibroids is an effective strategy for preventing the development of parasitic fibroids. Parasitic fibroids manifests in less than 1% of cases, typically following morcellation. Some viable fragments of fibroids will implant and continue to proliferate [11].

It is noteworthy that residual fibroids occurred in four cases in the electrotomy group in this study. This discrepancy may be attributed, at least in part, to the volume of fibroids in the electrotomy group exceeded that of the cold instrument group. While the data did not reach statistical significance, the maximum volume of fibroid in the electrotomy group was notably larger than that in the cold instrument group. Furthermore, after electrotomy of the fibroid till it is level with the uterine wall, the subsequent removal of the remaining fibroid, which is concealed within the myometrium, poses a significant challenge. In the study conducted by Murakami et al., complete removal of submucosal fibroid was achieved in only 57.1% of patients through a single surgical procedure. The study revealed that the probability of complete removal of the lesions in a single operation increased with the thickness of the free myometrial margin and decreased with an increase in the size of the fibroid and the volume of the fibroid within the myometrium [12]. Cold instruments uniquely suited for grasping the remaining myoma within the myometrium. Spoon forceps can be used to bluntly separate the myoma from the normal tissue, and then Allis forceps can be used to grasp the myoma, rotate it, and remove it, which helps to reduce the incidence of residual fibroid.

There was no statistically significant difference in the incidence of intraoperative and postoperative complications between the two groups. These findings suggest that cold instruments for hysteroscopic myomectomy are safe and effective.

## Conclusion

The results of this study confirm that cold instruments for hysteroscopic myomectomy seem to be a safe and effective surgical procedure, which is better than electrotomy with regard to complete removal of submucosal fibroids. Moreover, cold instruments for hysteroscopic myomectomy have no electrothermal damage to normal endometrium, which is favorable for pregnancy as soon as possible after surgery.

## Data Availability

No datasets were generated or analyzed during the current study.
